# NASA-Approved Rotary Bioreactor Enhances Proliferation of Human Epidermal Stem Cells and Supports Formation of 3D Epidermis-Like Structure

**DOI:** 10.1371/journal.pone.0026603

**Published:** 2011-11-09

**Authors:** Xiao-hua Lei, Li-na Ning, Yu-jing Cao, Shuang Liu, Shou-bing Zhang, Zhi-fang Qiu, Hui-min Hu, Hui-shan Zhang, Shu Liu, En-kui Duan

**Affiliations:** 1 State Key Laboratory of Reproductive Biology, Institute of Zoology, Chinese Academy of Sciences, Beijing, China; 2 Graduate School, Chinese Academy of Sciences, Beijing, China; University of Nebraska Medical Center, United States of America

## Abstract

The skin is susceptible to different injuries and diseases. One major obstacle in skin tissue engineering is how to develop functional three-dimensional (3D) substitute for damaged skin. Previous studies have proved a 3D dynamic simulated microgravity (SMG) culture system as a “stimulatory” environment for the proliferation and differentiation of stem cells. Here, we employed the NASA-approved rotary bioreactor to investigate the proliferation and differentiation of human epidermal stem cells (hEpSCs). hEpSCs were isolated from children foreskins and enriched by collecting epidermal stem cell colonies. Cytodex-3 micro-carriers and hEpSCs were co-cultured in the rotary bioreactor and 6-well dish for 15 days. The result showed that hEpSCs cultured in rotary bioreactor exhibited enhanced proliferation and viability surpassing those cultured in static conditions. Additionally, immunostaining analysis confirmed higher percentage of ki67 positive cells in rotary bioreactor compared with the static culture. In contrast, comparing with static culture, cells in the rotary bioreactor displayed a low expression of involucrin at day 10. Histological analysis revealed that cells cultured in rotary bioreactor aggregated on the micro-carriers and formed multilayer 3D epidermis structures. In conclusion, our research suggests that NASA-approved rotary bioreactor can support the proliferation of hEpSCs and provide a strategy to form multilayer epidermis structure.

## Introduction

Skin is one of the major organs of the body and considered as the primary protective barrier against the external environment. Adult skin is composed of two tissue layers: the stratified epidermis and the thick layer of collagen-rich dermal connective tissue. The epidermis, consisting of keratinocytes with variable degrees of differentiation,is constantly maintained by the population of self-renewing epidermal stem cell [1]. In addition, epidermal stem cells are deeply involved in tissue regeneration, wound healing, and neoplasm formation [2], [3].

The skin is susceptible to different injuries and diseases. Much attention has been given to patients with large-scale skin injuries such as severe burn or scald [4]. Tissue-engineered skin has been approved by the Food and Drug Administration in USA for use in wound healing, but the clinical results are far from satisfaction [5]. The application of this technique or method in therapy is presumably limited by the cultured epidermal and dermal autografts [6], [7]. Therefore, it is critical to improve the approaches to the isolation and culture of epidermal stem cells for their clinical utilization. Meanwhile, it remains a challenge for clinical application to improve the effect of wound healing and create a physiological three-dimensional (3D) tissue skin structure using EpSCs *in-vitro* before implantation.

Several studies showed that rotary bioreactor as a tool could influence major cellular events such as differentiation, proliferation, viability and cell cycle [8]. Recent studies have shown that rotation culture promotes the proliferation and viability of human periodontal ligament stem cells [9] and human mesenchymal stem cells [10]. Biomechanical force plays an important role to promote embryonic hematopoiesis [11]. Also some studies indicated that 3D clinostat for cell culture suppresses the differentiation of human osteoblast cells [12], human hematopoietic progenitor cells [13] and rat myoblasts [14]. More recently, simulative microgravity culture conditions were successfully used to culture mouse embryonic stem cells in feeder-free, serum-free media and LIF (Leukemia inhibitory factor) -free systems, [15] and to maintain the undifferentiated state and enhance the neural repair potential of bone marrow stromal cells [16]. We hypothesize that rotation cell culture system is also a feasible way to study human epidermal stem cells (hEpSCs) on the aspect of proliferation and differentiation. But to date, there are few related reports about this aspect.

Rotating cell culture system (RCCS) is a cell culture device made by NASA to simulate microgravity condition. It is also a 3D dynamic culture system for cell growth [17]. This culture system seems to be ideal for overcoming some drawbacks of static culture, because the rotational motion can prevent sedimentation, and create a suspension culture environment and enhance cell-cell interactions. Several researches showed that RCCS contribute to cellular aggregation, intercellular adhesion and formation of 3D cell clumps [18].

In this study, we enriched hEpSCs from 1–5 year old children foreskins according to cell size and collagen type IV adhesiveness method established in our group [19]. Isolated hEpSCs were expanded as previously described [20] and expanded cells were seeded on cytodex 3 micro-carriers and cultured in RCCS for 15 days. The proliferation and differentiation of cells were investigated under the same conditions. We found that rotary bioreactor enhances the proliferation and viability of hEpSCs in the first 10 days. In addition, rotation culture extenuates the differentiated state of hEpSCs compare with static culture. Furthermore, hEpSCs cultured in RCCS inclined to aggregate on the micro-carriers and form multilayer 3D epidermis-like structures. Our data suggest that NASA-approved rotary bioreactor may provide ideal approaches to creating a 3D epidermis tissue.

## Results

### Indentification of hEpSCs by colony forming efficiency, proliferative capacity and marker expression

**Figure 1:**
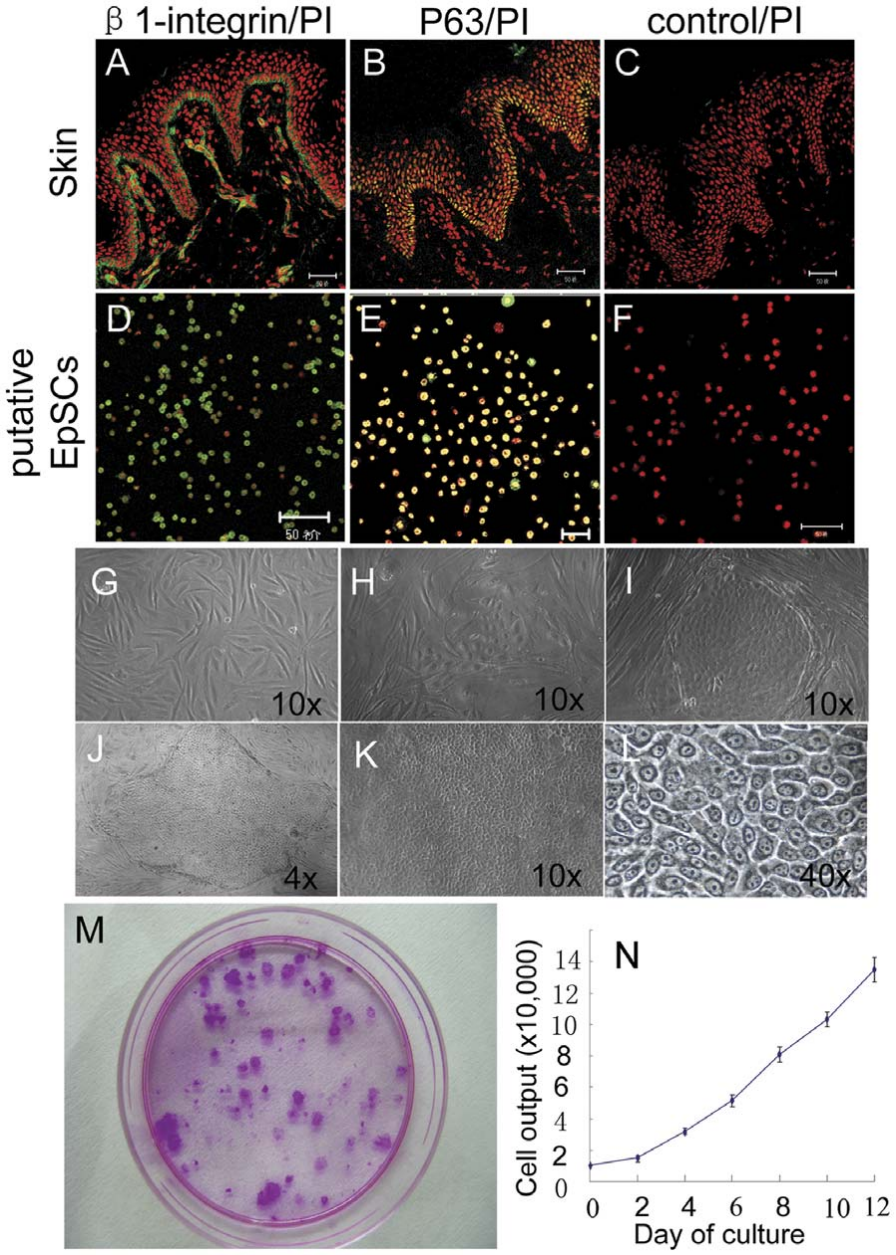
Putative stem cell marker expression in separated keratinocyte and probability of colony formation of separated cells. Expression of the β1-integrin and p63 protein (green) in human foreskin of skin (A, B) and in putative hEpSCs after isolated (D, E), which show high strong green fluorescent in cell membrane, cell nucleus. The image of feeder cells treated by mitomycin C (G), the colonies emerged on day 4 (H), 8 (I), and 12 (J–L) after seeding respectively. Visual colonies were observed at 12 day of culture (M). Growth curves of hEpSCs derived from 1×10^4^ cells (N).

To evaluate the cell growth capacity of putative hEpSCs isolated from children's foreskin, growth curve of hEpSCs was examined, and the colony formation efficiency (CFE) was counted. Immunofluorescence staining results revealed that almost all of the isolated cells expressed high level of β1-integrin and p63 protein (Fig.1 D, E), in accord with their high expression in the epidermal basal layer of skin (Fig.1A, B). The feeder cells were treated by mitomycin C (Fig.1G); and the colonies grew well, and emerged on day 4 (Fig.1H), 8 (Fig. 1I), and 12 (Fig. 1J–H) after cultured, respectively. The growth curve indicated that the total number of cells increased 12 folds after 12 days of culture (Fig. 1N). In addition, large colonies were formed and macroscopic after 12 days of growth with a colony formation efficiency (CFE) of 25% (Fig. 1M).

**Figure 2:**
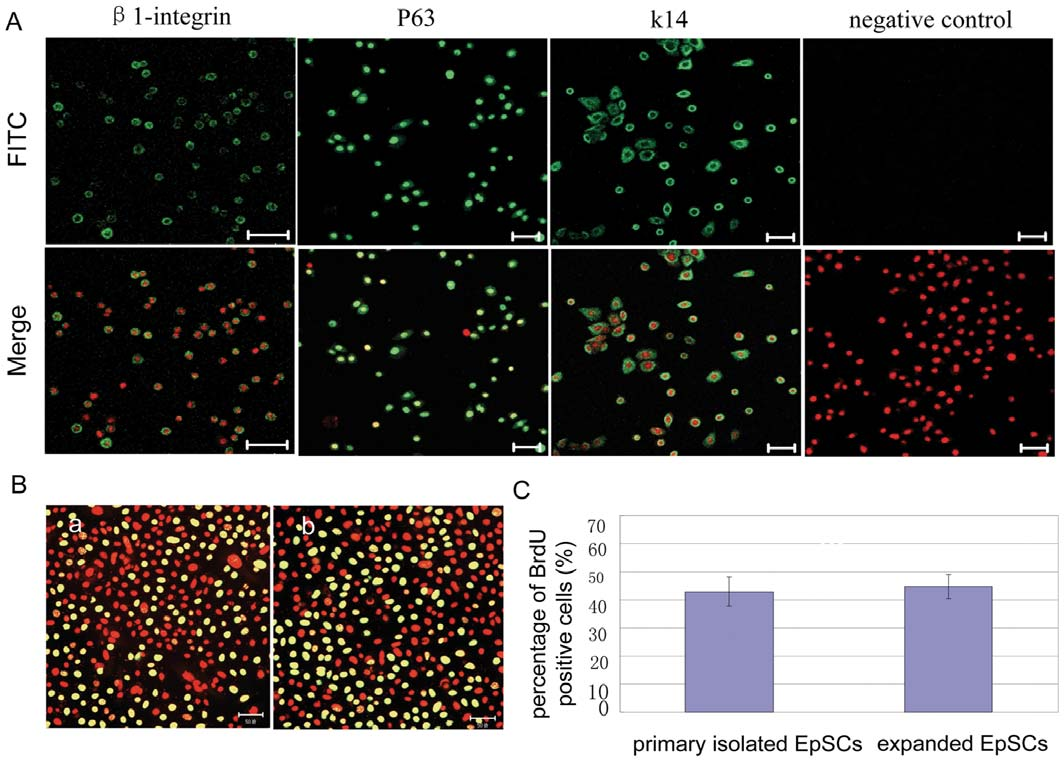
Expression of Putative Epidermal Stem Cell Marker and remained highly proliferate capacity when were expanded *in vitro*. (A) Expression of β1-integrin, p63 and k14 in hEpSCs cultured *in vitro* for 12 days after 3 passages. (B) BrdU positive cells were observed in separated cells (a) and expanded cells (b). (C) BrDU positive cells were quantitatively measured from three independent experiments and 200 cells in ten fields per case. Mean ± s.e. Bar, 50 µm (*, p> 0.05).

Before rotation culture experiments, to test whether these expanded cells still maintain characteristics of hEpSCs, we identified the expressions of hEpSCs markers after cell expanded. No significant differences of the expression level for β1-integrin, p63 or K14 were found between the isolated primary hEpSCs and the colony cells passageed three times from initial hEpSCs, and the majority of expanded hEpSCs were positive for these markers (Fig.2A). The percentage of β1-integrin, P63 and K14 positive cells is 98 ± 5%, 100 ± 2% and 100 ± 5%, respectively. Furthermore, BrdU incorporation assay indicated that these cells still maintained highly proliferative ability (Fig.2B, C). These results indicated that putative epidermal stem cell isolated from the human foreskin could be successfully propagated in our culture system for maintaining hEpSCs marker and supporting highly proliferative capacity.

### Generation of three-dimensional epidermis-like tissue in RCCS

**Figure 3:**
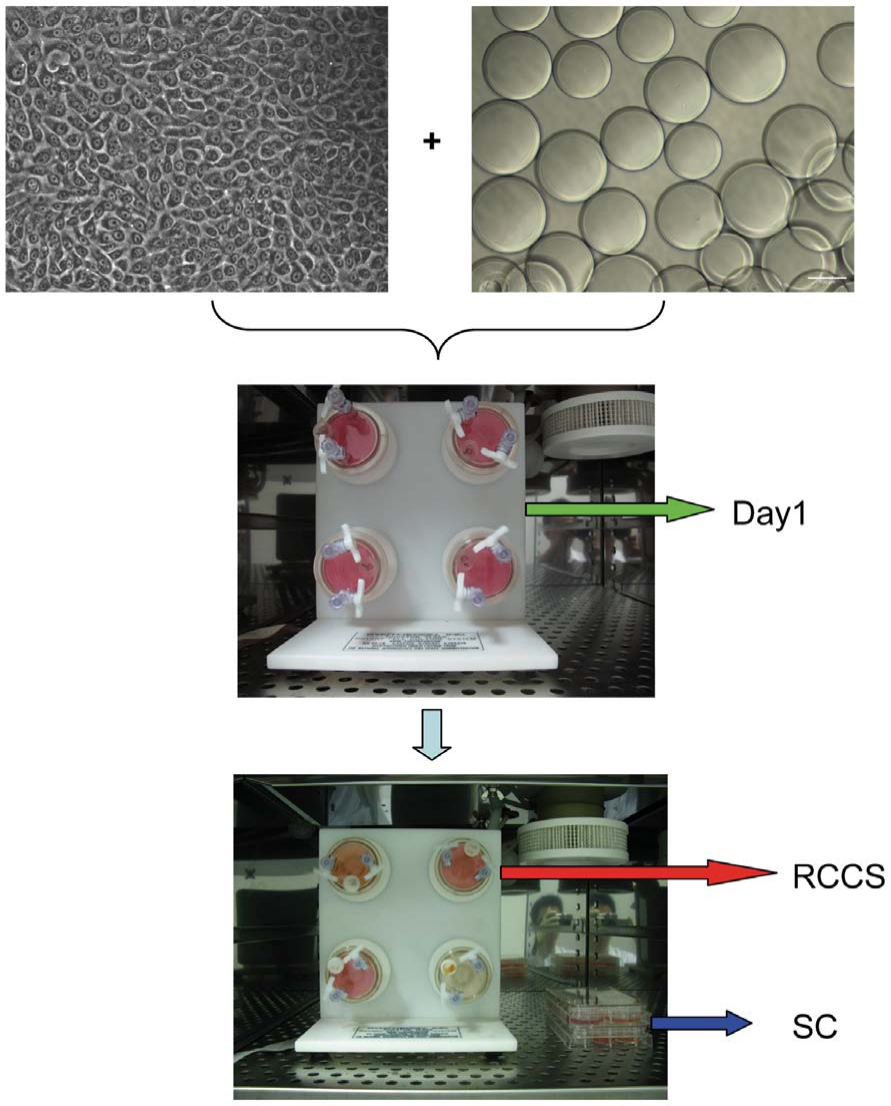
Schematic illustration of our experiment used to culture hEpSCs on cytodex 3 under static culture and RCCS. hEpSCs had been expanded for at least three passage on feeder cells before loaded into RCCS. The vessel was installed onto the RCCS rotating in a clockwise direction at a speed of 12 rpm in the first day. After cells adhered to the micro-carries 24 h later, they were randomly allocated to the the RCCS group and the static culture group.

**Figure 4:**
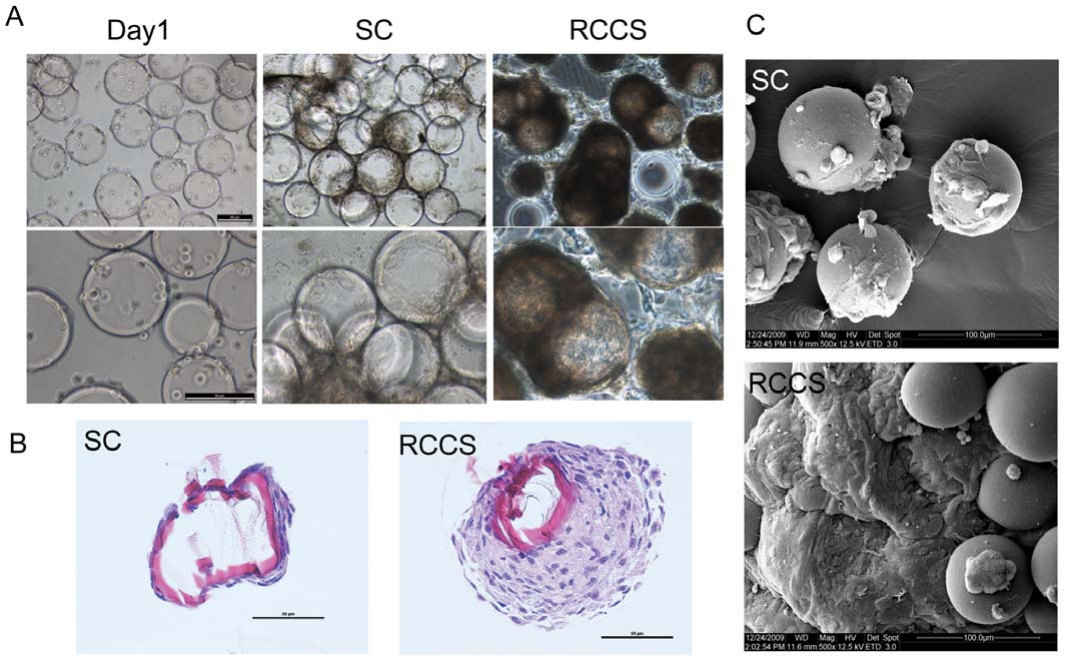
Micrographs of hEpSCs grown on Cytodex 3 micro-carriers under rotation culture and static culture. (A) Morphology of hEpSCs under condition of RCCS at day 1 (left panel) and day 10 of culture (right panel) and under condition of static culture (SC) at culture of day 10 (middle panel), respectively. The cells were being growing on micro-carrier beads and forming 3-D epidermis like structures. (B) The Morphology of the slices of micro-carriers which hEpSCs grew around cultured in SC and RCCS conditions (H&E) at the 10^th^ day of culture. hEpSCs of SC group formed monolayer structure (left panel). In contrast, the RCCS group showed that hEpSCs grew into multicellular structures on micro-carriers (right panel). (C) Scanning electron microscopy of the structure, cultured under RCCS and SC at day 10. Bars = 50 µm.

To examine the effect of rotary culture on the growth of hEpSCs, colony cells were trypsinized, and seeded in rotary bioreactor together with micro-carrier beads for 1 day, which allowed cells to attach to the surface of the beads. Then the sample was divided into two groups, including rotation culture group in RCCS and static culture group on 6-well cell culture plate (Fig. 3). Fig. 4A showed the micrographs of hEpSCs grown on cytodex 3 micro-carriers for 1 day and 10 day after culture under the conditions of rotation culture and static culture. The cells in rotary bioreactor were prone to accumulate on the micro-carrier beads, and cluster of cells or 3D aggregates were formed (Fig. 4A, right panel), while cells in 6-well cell culture plate appeared monolayer sheet structure on the surface of micro-carrier beads (Fig. 4A, middle panel). In rotation culture group, there were a large number of cells aggregated over micro-carriers beads forming 3D tissue-like epidermis structure. Moreover, the gaps among micro-carriers beads were covered with cells compactly. Conversely, there were few cells growing on the surface of micro-carriers beads in static culture. Similar result or structure was observed through H&E staining (Fig.4B). A few monolayer cells could be observed on the micro-carriers in the static culture group (Fig.4B, left panel), whereas cells in the rotation culture group displayed a 3D multicellular spheroids (Fig.4B, right panel), which were morphologically similar to the 3D structure of epidermis *in vivo*.

To further examine the morphology of 3D aggregates after 10 days in rotary bioreactor and 6-well cell culture plate, samples were also were observed by SEM (Fig.4C). In static culture group, there were only a single layer of epidermal cells on the surface of the micro-carrier beads (Fig.4C, the upper panel), whereas SEM of rotation culture group displayed multilayers, typical of 3D epidermis structure (Fig.4C, the bottom panel). In addition, we also found that the bigger clusters or 3D aggregates of cells at 10 day culture under rotation culture condition (data not shown).

### Promotion of proliferation and inhibition of differentiation in three-dimensional cell culture of RCCS

**Figure 5:**
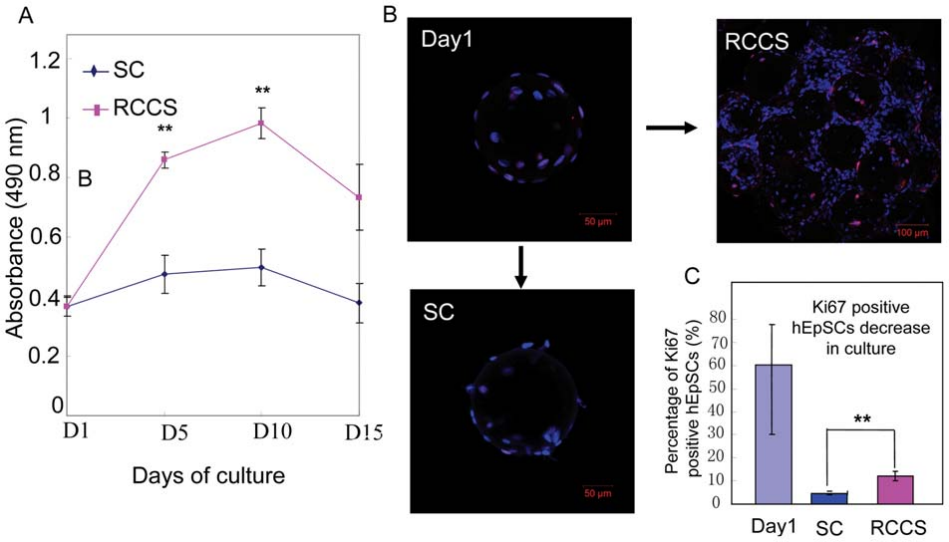
Effects of the rotated three dimensional cell culture system on the proliferation of hEpSCs. (A) The number of viable hEpSCs in proliferation or cytotoxicity assays under RCCS and SC culture conditions. (B) Expression of ki-67 in hEpSCs cultured at day 10 cultured under RCCS condition and static culture. (C) Ki67 positive cell percentages in hEpSCs cultured at day 10 cultured under RCCS condition. Mean ± s.e.**, p<0.01.

**Figure 6:**
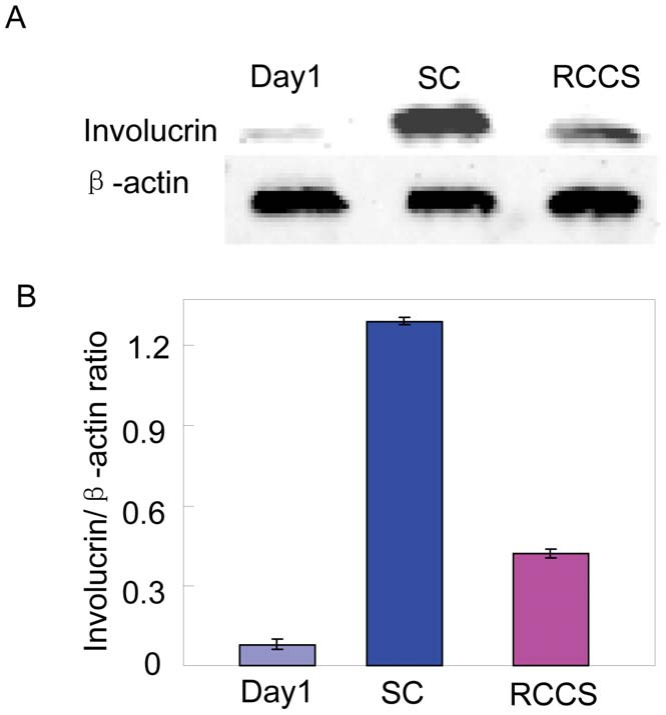
Effects of the rotated three dimensional cell culture system on the expression levels of involucrin. (A) Protein was extracted, and immunoblot was used to detect the specific antibody to involucrin at the 10^th^ day of culturing, (B) densitometry values from western blot analyses of involucrin proteins in the cells of day1, RCCS and SC cultured hEpSCs, respectively. Mean ± s.e. **, p<0.01.

The effect of rotation culture on proliferation of hEpSCs was investigated through MTS assay. As shown in Fig. 5A, rotation culture condition remarkably promoted the cell proliferation potential in culture at day 5 and 10 compared with that in static culture condition. We further examined cell proliferation and differentiation of hEpSCs by immunocytochemistry assay and western blot. Ki67 is a marker of proliferation [21], and involucrin is a marker of terminal differentiation of hEpSCs [22]. Results showed that the differences for percentage of ki67 positive cells were statistically significant between rotation culture and static culture in the culture for 10 days (t test, *P*<0.01). The percentage of ki67 positive cells per aggregate was about 10% under rotation culture condition (Fig.5C). On the contrast, for static culture group, there was very low percentage of ki67 positive cells (Fig.5C), suggesting that rotation culture seemingly support the proliferation of hEpSCs under a feeder free culture condition. Conversely, we compared the expression of involucrin in rotation culture with that in static culture at protein level. These results showed that the expression of involucrin in rotation culture was significantly lower than that in static culture on day 10 (Fig.6A). However, we did not observe visible variation of the involucrin expression in cells of cultured at day 1. (Fig.6A, B). These results demonstrate that RCCS may provide a condition to promote cells proliferation and maintain the low differentiation state forming a mutilamellar of epidermis.

## Discussion

Clinical applications of tissue-engineered skin substitute represent a strategy for wound healing [23], and presently cultured autografts are still considered to be the preferred candidate for wound healing. Currently, two major questions are confronted in wound repair and skin regeneration research: one is to isolate and propagate epidermal stem cells *in vitro*; the other is to create a physiological 3D tissue structure *in vitro* before transplantation [24]. However, there are still many technical challenges to be overcome to attain a highly efficient isolation/enrichment of hEpSCs and the ability of culturing the cells to be a natural 3D structure [23].

Firstly, we have described a procedure for the isolation and culture of hEpSCs derived from human foreskin using a previously described method with modifications. In our system, epidermal stem cells isolated from the human foreskin could be successfully propagated in vitro, maintaining hEpSCs marker and supporting highly proliferative capacity.

Secondly, studies were carried out with a NASA rotary bioreactor to investigate the effect of rotational culture on proliferation of hEpSCs. Interestingly, we found that rotational culture seemed to offer several advantages for hEpSCs growth, particularly for the generation of 3D epidermal aggregates under feeder-free culture condition. According to the results of MTS assay, hEpSCs in RCCS proliferated at day 5 and 10 of culture, while cells in static culture condition exhibited insignificant changes on the surface of micro-carriers. Moreover, the measurement of Ki67, a commendable marker of proliferation, was included in our experiment. The measurement indicated that Ki67 positive cells could be observed by day 10 of culture in the RCCS. However, almost no Ki67 positive cells were observed by day 10 of static culture in the 6-well plate. These results indicated that the rotation culture conditions produced by RCCS were superior to static culture for hEpSCs under feeder-free culture condition. Interestingly, similar results were observed for human periodontal ligament stem cells, osteoblast like cells and rat marrow stromal cells after culture in a rotating wall vessel bioreactor [9], [25], [26].

Further, to explore the mechanism of hEpSC proliferation and 3D structure formation under rotational culture condition. The effect of cell differentiation was considered chiefly. Involucrin, a known maker for terminal differentiation of keratinocytes [27], was examined in this study. Based on our western blot results, by day 10 of culture, weak expression of involucrin was detected in cells cultured under RCCS, whereas strong expression of involucrin was detected in cells cultured under static culture conditions. Our results suggest that RCCS could potentially provide a favorable environment to inhibit the differentiation of hEpSCs. This is consistent with previous findings in which the rotating wall vessel bioreactor has been shown to provide a culture platform for the repression of terminal differentiation in many cell types [28], [29].

In our culture system, cytodex 3, prepared with a cover of a thin layer of denatured collagen chemically coupled to a matrix of cross-linked dextran, was used in this study. Previous studies have indicated that cytodex 3 could support the growth of a wide variety of cells [30]–[32]. Importantly, compared with other culture methods, cytodex 3 provides the possibility for the quick expansion of cells on the large surface of spherical carriers thereby avoiding further enzymatic treatment before transplantation [33]. Moreover, we suggested that the rotating speed of RCCS should be adjusted at a low level of about 12 rpm on the first day, because under these conditions the visible cells and cytodex 3 aggregates formed a fluid orbit within the vessel, exhibitting continual free fall, and did not make contact with the wall of the vessel, resulting in an increase in the number of cells attached to the micro-carriers. On the second day, the rotating speed was increased to 22 rpm and held constant during the additional 14 days of culture.

The fundamental process for the creation of a 3D epidermis is cellular proliferation and differentiation to obtain a stratified, squamous epithelium. Epidermal differentiation is a common process for keratinocytes during the formation of the mature epidermis tissue, and some terminal differentiation markers appear in the cells at the middle or later stages of differentiation [34]. Our studies showed that the expression of involucrin was significantly lower in cells of the RCCS group than that of the static culture group on day 10 of culture. So, our data suggest that a rotational 3D culture may provide a beneficial environment for epidermis development, as the keratinocytes can interact with each other in a microenvironment that allows differentiation to proceed in a similar pattern to skin development *in vivo*. This viewpoint was also suggested by Lewis and Duray, who discovered that tissues could be cultured in rotating wall vessel bioreactor as for organ culture and maintain their overall structure comparable to the original explants [35], [36].

To summarize, our results demonstrate that RCCS may provide an ideal physical and chemical environment to guide hEpSCs proliferation and provides an acceptable culture model to assemble 3D multilayer epidermis tissue. Moreover, this dynamic system may have a putative value as screening tool of skin drug research in the future.

## Materials and Methods

### Isolation of epidermal stem cells and cell expansion

Human foreskin samples were derived from voluntary circumcisions with informed consents and the protocol was approved by the Ethical Committee of the Institute of Zoology, Chinese Academy of Sciences. Human epidermal stem cells (hEpSCs) were isolated and expanded using our previous described method [19], [20]. Briefly, Skin from children' foreskin aged 1 to 5 years were isolated based on their rapid adherence to collagen type IV and their small cell size. Then separated cells were expanded on MMC treated human dermal fibroblast feeder cells using FAD complete culture medium (DMEM: Ham's F-12/3∶1), containing with 10% FCS (Gibco), 5 µg/ml transferrin (T3309, Sigma), 10 ng/ml EGF (100-15, Peprotech), 0.18 nM adenine (A2786, Sigma), 5 µg/ml insulin (I5500, Sigma), 0.4 µg/ml cholera toxin (C8052, Sigma) and 0.5 µg/ml hydrocortisone (H6909, Sigma) and maintained at 37°C in a humidified 5% CO2 incubator. Cells were observed every day and the medium was changed every three days.

To determine the growth curve, the hEpSCs (1×10^4^ cells) were plated into 6 cm dishes, the cells outputs were counted every two days for two weeks. In addition, after two weeks, the colonies of were fixed with 4% formaldehyde and stained with 1% rhodamine B (R6626, Sigma) and the colony formation efficiency were counted. Only the colonies that contained more than 32 cells were accounded. To compare the proliferation between initial isolated EpSCs and expanded EpSCs, bromodeoxyuridine (BrdU, B9285 Sigma) were added to the media at a final concentration of 20 µM for 12 h in accordance to our previous report [37].

### Micro-carrier preparation, seeding and culture

Cytodex 3, a micro-carrier used in this study, was treated according to the manufacturer protocol. In brief, cytodex 3 (C3275, Sigma) were rehydrated in PBS at room temperature for 3 hours, washed 3 times and autoclaved for 20 min, then coated by collagen type IV (100 µg/ml, C5533, Sigma) overnight and soak in PBS at 4°C. Before cell seeding, micro-carriers were rinsed once with FAD complete culture media.

For culture, 5×10^3^ cells/ml EpSCs and 1 mg/ml micro-carriers were inoculated to 10 ml culture vessel of RCCS (Synthecon, Inc., Houston, TX, U.S.A) with 10 ml FAD complete culture media. The vessel was installed onto the RCCS rotating in a clockwise direction at a speed of 12 rpm in the first day. After cells adhered to the micro-carries 24 later, they were randomly allocated to the two groups, the static culture group and the RCCS group. In RCCS group, cells were seeded in vessel of RCCS and the rotational speed was then changed to 22 rpm until the end of 15 days culture. In static culture group, cells were cultured in 6-well plates. The medium was changed every 5 days and the cells were observed at that times.

### Growth and proliferation measurements

Since EpSCs had been cultured in the RCCS, the changes in number and viability of cells were checked by MTS assay at day 1, 5, 10 and 15 according to the manual instructions. Briefly, 20 µl of CellTiter 96 AQueous One Solution Reagent (G-3582, Promega) was added into each 96-well plate containing the samples in 100 µl of culture medium, and then plate was incubated for 4 h at 37°C in humidity, 5%CO_2_ atmosphere. The absorbance was measured at 490 nm. The parallel assay was used to EpSCs static cultured in 6-well plates as the static culture group.

### Histological staining

After cultured for 10 days, samples with monolayer cells in static culture group and multilayer cells in rotation culture group were fixed with Bouin's solution for 10 h. Warm liquid agar (1.5%) was added into a plastic plate containing the samples of micro-carrier-cells mixture, then cooled to solid form. The agar blocks were fixed in 75% ethanol for about 30 min, and embedded by paraffin. Then five micrometer thick sections stained with hematoxylin and eosin (H&E) were prepared for morphological observation. For static culture group, samples were also treated and examined with the same methods.

### Scanning electron microscopy sample preparation and microscopy

The 3D cell-micro-carrier aggregates that were cultured for 10 days were washed with PBS and fixed in fresh fixative (2.5% glutaraldehyde (SPI-Chem)) in PBS for 24 h at 4°C. After being washed with PBS for three times, they were then fixed with 1.0% osmium tetroxyde (Electron Microscopy Sciences, Hatfield, PA, USA) in PBS for 1 h at room temperature. Samples were then dehydrated in a six-step series of ethyl alcohol, gradually grade at 30%, 50%, 70%, 80%, 90% and 100%. After treated with iso-amylacetate for 5 h, the samples were dried to critical point and coated with gold. Finally, samples were imaged with Scanning Electron Microscopy (SEM, FEI Quanta-200). Static culture of epidermal cells was parallel treated and examined with the same methods.

### Immunohistochemistry and immunocytochemistry staining

Frozen sections, cells or micro-carrier-cell samples were fixed with 4% paraformaldehyde (PFA) at room temperature for 30 min respectively, and then treated with 5% bovine serum albumin (BSA)/PBS. After incubated with different primary antibodies (diluted in PBS with 1% BSA) overnight at 4^⌊^, samples were then rinsed three times followed by incubating with FITC-conjugated or rhodamine-labeled secondary antibodies (1∶100, Zhongshan Biotechnology) at 37^⌊^ for 1 h. After three washes, nuclei were stained with 10 µg/ml propidium iodide (Sigma) or with 1 µg/ml Hoechst 33342 (Sigma) for 10 min at room temperature before viewed under laser scanning confocal microscope (Zeiss, LSM 710 Meta, Germany). Primary antibodies used were listed in Table 1.

**Table 1:** Primary antibodies used in immunostaining.

Antibodies	Dilution	Manufacturer and Code number
β1-integrin	1∶200	Santa Cruz, sc-9970
p63	1∶200	Santa Cruz, sc-9431
cytokeratin 14	1∶200	Santa Cruz, sc-17104
Ki67	1∶200	Thermo Scientific, 9106-S

### Western blot analysis

The expression level of involucrin was determined by Western blot. The samples after 10 days of culture under rotary bioreactor and 6-well dish were lysed in detergent lysis buffer (SBS Genetech, Beijing). A total of 20 µg protein extracts was separated by 15% SDS–PAGE, then electron transferred onto a PVDF membrane (Amersham, Arlington Heights, IL). The membrane was blocked with 5% nonfat milk in tris-buffered saline containing 0.4% Tween 20, and then incubated with primary antibody: anti-involucrin (1∶2000, sc-15223, Santa Cruz Biotechnology) and anti-β-actin (1∶1000, sc-47778, Santa Cruz). After incubation with IR Dye 800 (1∶5000, LI-COR, Inc., Lincoln, NE, USA) labeled secondary antibody, the membranes was subjected to the Odyssey imaging system (LI-COR Biosciences).

### Statistical analysis

All experiments were repeated 4 times. Results were presented as the mean ±s.e of 4 separate experiments. Statistical differences were evaluated by Student's t-test analysis or variance (ANOVA).
